# Why Is the Universe Not Frozen by the Quantum Zeno Effect?

**DOI:** 10.3390/e27060652

**Published:** 2025-06-18

**Authors:** Antoine Soulas

**Affiliations:** 1 Faculty of Physics, University of Vienna, Boltzmanngasse 5, 1090 Vienna, Austria; antoine.soulas@univie.ac.at; 2 IQOQI Vienna, Austrian Academy of Sciences, Boltzmanngasse 3, 1090 Vienna, Austria

**Keywords:** quantum Zeno effect, decoherence, mathematical physics

## Abstract

We built a discrete model that simulates the ubiquitous competition between the free internal evolution of a two-level system and the decoherence induced by the interaction with its surrounding environment. It is aimed at being as universal as possible, so that no specific Hamiltonian is assumed. This leads to an analytic criterion, depending on the level of short time decoherence, allowing one to determine whether the system will freeze due to the Zeno effect. We checked this criterion on several classes of functions which correspond to different physical situations. In the most generic case, the free evolution wins over decoherence, thereby explaining why the universe is indeed not frozen. We finally make a quantitative comparison with the continuous model of Presilla, Onofrio and Tambini, based on a Lindblad’s master equation, a find good agreement at least in the low coupling regime.

## 1. Introduction

The Zeno effect typically occurs when a quantum system is repeatedly measured: if the time interval between two successive measurements tends to 0, the evolution of the system becomes frozen. The main reason is that, in quantum mechanics, the general short time evolution is quadratic, i.e.,

|〈Ψ|Ψ(t)〉|2=|〈Ψ|e−iH^t|Ψ〉|2=1−Vt2+Ot4,

where V≡Var|Ψ〉(H^)=〈Ψ|H^2|Ψ〉−〈Ψ|H^|Ψ〉2 (we take ℏ=1). Hence, if *n* projective measurements along |Ψ〉 are performed during a fixed time interval *T*, the probability $p_{n}$ that all measurements give the outcome |Ψ〉 is, at leading order:

$$p_{n}≃{\left(1-V{\left(\frac{T}{n}\right)}^{2}\right)}^{n}\underset{n\to +\infty }{⟶}1.$$

Note that, to obtain this limit, one has to neglect the higher order terms, as is usually done in the standard presentations of the Zeno effect (Section 3 of [1] and Section 3.3.1.1 of [2]). Rigorously speaking, this is an additional assumption, because when developing the expression ${\left(1-V{\left(\frac{T}{n}\right)}^{2}+O\left(\frac{1}{n^{4}}\right)\right)}^{n}$, the number of $O\left(\frac{1}{n^{4}}\right)$ actually depends on *n*.

In the spirit of the theory of decoherence, one might wish to become rid of the ill-defined notion of (ideal) projective measurement. Since a measurement is nothing but a particular case of interaction with an environment that entails strong decoherence of the system in the measured basis, it is tempting to ask what level of decoherence is required to freeze a system. For example, a particle in a gas is continuously monitored (∼measured) by its neighbors, yet the gas manifestly has an internal evolution—and so does the universe in general. It is not obvious a priori whether quantum mechanics actually predicts that the universe is not frozen.

This question has already been addressed by examining the continuous dynamics of the pair system–environment for a relatively generic Hamiltonian [3]. The Zeno limit is recovered for strong interaction, and Fermi’s golden rule is recovered in the limit of small interaction. “The model shows that the coupling to the environment leads to constant transition rates that are unaffected by the measurement if the coupling is ‘coarse enough’ to discriminate only between macroscopic properties. This may in turn be used to define what qualifies a property as macroscopic: it must be robust against monitoring by the environment” (Section 3.3.2.1 of [2]). Similarly, the master equation for the motion of a mass point under continuous measurement indicates that the latter is not slowed down because the Ehrenfest theorems are still valid. “This may be understood as a consequence of the fact that, for a continuous degree of freedom, any measurement with finite resolution necessarily is too coarse to invoke the Zeno effect” (Section 3.3.1.1 of [2]).

In addition, Presilla, Onofrio, and Tambini have studied how the continuous monitoring of a two-level system affects its time evolution and its coherence based on a Lindblad’s master equation (Section IV of [4]). They have in particular confronted their results to that of a historical experimental test of the Zeno effect [5] and were able to obtain a better fit of the data than the naïve approach (assuming perfect von Neumann collapses) by taking into account the finite duration of the measurements. We will come back to this approach in Section 5 to make a quantitative comparison between the latter’s results and ours.

Another interesting model is that of Sections 8.3 and 8.4 of [6]. It may at first sight seem puzzling that an unstable nucleus continuously measured by a Geiger counter can actually decay. Indeed, if the measurement is treated as an ideal projective one, the nucleus should continuously be projected onto a non-decayed state. However, as soon as the decoherence process is not supposed immediate anymore (even as short as $10^{-16}$ s, see Equation (8.45) in [6]), the deviation from the expected exponential decay is shown to be negligible.

Although these models are already convincing, our aim is to make a new contribution to this topic by explaining why the vast majority of physics is not affected by the quantum Zeno effect, the latter being detectable only in some very specific experimental setups. Our model also formalizes the competition between free evolution (no information leaking to the rest of the world) and decoherence (interaction with the environment), but differs from the previous ones in two respects: its mathematical structure is discrete and it does not assume anything about the form of the Hamiltonian, so as to be as universal as possible. The use of a discrete framework is consistent with the approach adopted in many mathematical studies on the quantum Zeno effect (see [7] and references therein).

## 2. The Model: Free Evolution vs. Decoherence

Having in mind the fact that continuous degrees of freedom are less prone to the Zeno effect (recall the previous quote from [2]), in order to explain why the universe is not frozen, it may suffice to check it on a two-level system. Our system of interest will therefore be a qbit, initially in the state |0〉 and monitored by an environment producing partial decoherence in the basis (|0〉,|1〉). We consider a fixed time interval *T*, divided into *n* phases of length $\delta =\frac{T}{n}$ dominated by the free evolution. This evolution takes the general form

$$\left\{\begin{array}{c} U_{\delta }|0\rangle =c_{=}^{0}\left(\delta \right)|0\rangle +c_{\neq }^{1}\left(\delta \right)|1\rangle \\ U_{\delta }|1\rangle =c_{\neq }^{0}\left(\delta \right)|0\rangle +c_{=}^{1}\left(\delta \right)|1\rangle , \end{array}\right.$$

where the coefficients satisfy ${\left|c_{=}^{0}\left(\delta \right)\right|}^{2}={\left|c_{=}^{1}\left(\delta \right)\right|}^{2}=1-V\delta^{2}+O\left(\delta^{4}\right)$ and ${\left|c_{\neq }^{0}\left(\delta \right)\right|}^{2}={\left|c_{\neq }^{1}\left(\delta \right)\right|}^{2}=V\delta^{2}+O\left(\delta^{4}\right)$ (in the sequel, we will drop the argument δ whenever the context is clear). As mentioned in the introduction, to stick to the standard derivations of the Zeno effect, we need to neglect all the higher order terms, so that we actually suppose ${\left|c_{=}^{0}\left(\delta \right)\right|}^{2}={\left|c_{=}^{1}\left(\delta \right)\right|}^{2}=1-V\delta^{2}$ and ${\left|c_{\neq }^{0}\left(\delta \right)\right|}^{2}={\left|c_{\neq }^{1}\left(\delta \right)\right|}^{2}=V\delta^{2}$.

**Figure 1:**
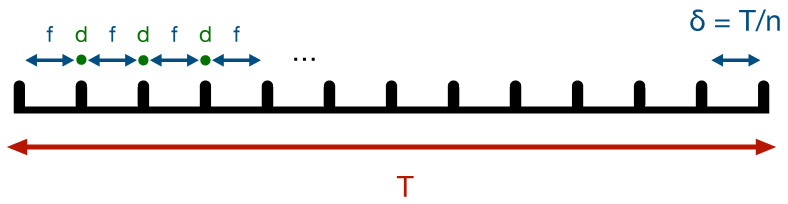
Alternating steps of free evolution (f) and decoherence (d).

After the i^th^ phase of free evolution, the system meets some neighboring environment $E^{i}$, initially in the state $|E_{\mathrm{init}}^{i}\rangle$, and becomes immediately entangled according to

$$\left\{\begin{array}{c} |0\rangle ⟶|0\rangle |E_{0}^{i}\rangle \\ |1\rangle ⟶|1\rangle |E_{1}^{i}\rangle , \end{array}\right.$$

where $\left|\langle E_{0}^{i}|E_{1}^{i}\rangle \right|\equiv \eta^{i}$ quantifies the level of decoherence induced by $E^{i}$, i.e., how well the environment has recorded the system’s state ($\eta^{i}=1$ means no decoherence, $\eta^{i}=0$ perfect decoherence). See Figure 1.

We henceforth suppose that $\eta^{i}\equiv \eta$ does not depend on *i* (taken as a mean level of decoherence), which amounts to assuming that the strength of the interaction is more or less constant over time. Finally, we also suppose that each environment $E^{i}$ is distinct from the others and non-entangled at the time it encounters the system.

Recalling that T=nδ, the relevant quantity to compute is the probability $p_{n}$ that, at the end of the time interval *T*, the system is still found in its initial state |0〉 and that all the successive environments have recorded 0.

**Proposition** **1.** *Neglecting all the higher order terms, we can write*

$$\begin{array}{c} p_{n}≃1-2\left[\frac{n}{2}+\left(n-1\right)\eta +\left(n-2\right)\eta^{2}+\ldots +\eta^{n-1}\right]V\delta^{2}. \end{array}$$

**Proof.**  The cases n=1 or 2 are easy to treat. Indeed, the successive iterations are as follows (*f* stands for the free evolution, and *d* for the decoherence step):

$$\begin{array}{cc} |0\rangle \equiv |\Psi_{0}\rangle & \overset{f}{⇝}c_{=}^{0}|0\rangle +c_{\neq }^{1}|1\rangle \\ \; & \overset{d}{⇝}c_{=}^{0}|0\rangle |E_{0}^{1}\rangle +c_{\neq }^{1}|1\rangle |E_{1}^{1}\rangle \equiv |\Psi_{1}\rangle \\ \; & \overset{f}{⇝}c_{=}^{0}\left[c_{=}^{0}|0\rangle +c_{\neq }^{1}|1\rangle \right]|E_{0}^{1}\rangle +c_{\neq }^{1}\left[c_{\neq }^{0}|0\rangle +c_{=}^{1}|1\rangle \right]|E_{1}^{1}\rangle \\ \; & \;\;=|0\rangle \left[c_{=}^{0}c_{=}^{0}|E_{0}^{1}\rangle +c_{\neq }^{0}c_{\neq }^{1}|E_{1}^{1}\rangle \right]+|1\rangle \left[c_{\neq }^{1}c_{=}^{0}|E_{0}^{1}\rangle +c_{=}^{1}c_{\neq }^{1}|E_{1}^{1}\rangle \right]\\ \; & \overset{d}{⇝}|0\rangle \left[c_{=}^{0}c_{=}^{0}|E_{0}^{1}\rangle +c_{\neq }^{0}c_{\neq }^{1}|E_{1}^{1}\rangle \right]|E_{0}^{2}\rangle +|1\rangle \left[c_{\neq }^{1}c_{=}^{0}|E_{0}^{1}\rangle +c_{=}^{1}c_{\neq }^{1}|E_{1}^{1}\rangle \right]|E_{1}^{2}\rangle \equiv |\Psi_{2}\rangle . \end{array}$$

The ${\left(|\Psi_{n}\rangle \right)}_{n\in N}$ seem to live in different Hilbert spaces only because we omit all environments ${\left(E^{i}\right)}_{i\geq n+1}$ with which the system is not entangled yet. Consequently, neglecting all the higher order terms yields

$$\begin{array}{cc} •\;p_{1} & ={\left|\langle 0E_{0}^{1}|\Psi_{1}\rangle \right|}^{2}={\left|c_{=}^{0}\right|}^{2}=1-V\delta^{2}\\ •\;p_{2} & ={\left|\langle 0E_{0}^{1}E_{0}^{2}|\Psi_{2}\rangle \right|}^{2}\\ \; & ={\left|c_{=}^{02}+c_{\neq }^{0}c_{\neq }^{1}\langle E_{0}^{1}|E_{1}^{1}\rangle \right|}^{2}\\ \; & ={\left(1-V\delta^{2}\right)}^{2}+\eta^{2}{\left(V\delta^{2}\right)}^{2}+2ℜ\left({\overset{¯}{c_{=}^{0}}}^{2}c_{\neq }^{0}c_{\neq }^{1}\langle E_{0}^{1}|E_{1}^{1}\rangle \right)\\ \; & ≃1-2\left(1+\eta \right)V\delta^{2}. \end{array}$$

The last step is not obvious and comes from the following argument. A priori, the quantity $ℜ\left({\overset{¯}{c_{=}^{0}}}^{2}c_{\neq }^{0}c_{\neq }^{1}\langle E_{0}^{1}|E_{1}^{1}\rangle \right)$ lies in $\left[-\eta V\delta^{2},\eta V\delta^{2}\right]$ up to a $O\left(\delta^{4}\right)$, but the coefficients of the matrix $U_{\delta }$ are not unrelated. Using the general parametrization of a 2×2 unitary matrix, $U_{\delta }=\left(\begin{array}{cc} c_{=}^{0} & c_{\neq }^{0}\\ c_{\neq }^{1} & c_{=}^{1} \end{array}\right)$ can be written as $\left(\begin{array}{cc} a & b\\ -e^{i\varphi }\overset{¯}{b} & e^{i\varphi }\overset{¯}{a} \end{array}\right)$. Moreover, for small δ (this approximation may be rough for the case n=2 but improves as *n* increases), $U_{\delta }\to 1$; hence, $\det\left(U_{\delta }\right)=e^{i\varphi }\to 1$ and $\overset{¯}{a}\to 1$. We also expect $\langle E_{0}^{1}|E_{1}^{1}\rangle$ to be close to the real number 1 (infinitesimal decoherence). Combining all this, ${\overset{¯}{c_{=}^{0}}}^{2}c_{\neq }^{0}c_{\neq }^{1}\langle E_{0}^{1}|E_{1}^{1}\rangle =-e^{i\varphi }{\overset{¯}{a}}^{2}{\left|b\right|}^{2}\langle E_{0}^{1}|E_{1}^{1}\rangle$ is close to be a real negative number, so its real part is approximately the opposite of its modulus.

In general, $p_{n}={\left|\langle 0E_{0}^{1}\ldots E_{0}^{n}|\Psi_{n}\rangle \right|}^{2}$ is the square modulus of a sum of terms of the form

$$z_{\alpha }^{b}=c_{\alpha_{1}}^{b_{1}}\ldots c_{\alpha_{n}}^{b_{n}}\;\langle E_{0}^{1}|E_{b_{1}}^{1}\rangle \ldots \langle E_{0}^{n}|E_{b_{n}}^{n}\rangle ,$$

where $\alpha =\alpha_{1}\ldots \alpha_{n}$ and $b=b_{1}\ldots b_{n}$ are words on the alphabets {=,≠} and {0,1}, respectively. The word *b* is entirely deduced from $\alpha_{1}\ldots \alpha_{i}$ according to

$$\begin{array}{c} b_{0}=0\;;\;b_{i}=\left\{\begin{array}{cc} b_{i-1}\; & \;\mathrm{if}\;\alpha_{i}\;\mathrm{is}\;=\;(\mathrm{state}\;\mathrm{preserved})\\ b_{i-1}+1\;\mathrm{mod}\;2\; & \;\mathrm{if}\;\alpha_{i}\;\mathrm{is}\;\neq \;(\mathrm{state}\;\mathrm{flipped}), \end{array}\right. \end{array}$$

with the additional requirement that $b_{n}=0$ (the system finally measured in state |0〉), so that α actually contains an even number of ≠. Note that only the indices *i* such that $b_{i}=1$ contribute non-trivially to the product of brackets, since $\langle E_{0}^{i}|E_{0}^{i}\rangle =1$.

We now use the fact that ${\left|\sum_{k}z_{k}\right|}^{2}=\sum_{k}{\left|z_{k}\right|}^{2}+\sum_{k<l}2ℜ\left(\overset{¯}{z_{k}}z_{l}\right)$ for all complex numbers ${\left(z_{k}\right)}_{k}$. In our case, the leading term is clearly ${\left|z_{=\ldots =}^{0\ldots 0}\right|}^{2}={\left|{c_{=}^{0}}^{n}\right|}^{2}={\left(1-V\delta^{2}\right)}^{n}≃1-nV\delta^{2}$, while all the other square moduli are of order $\delta^{4}$ or less because they contain at least two factors ${\left|c_{\neq }^{b_{i}}\right|}^{2}=V\delta^{2}$. Furthermore, repeating the above argument, the real parts can be approximately replaced by their opposite moduli (and this approximation is more accurate as *n* becomes larger). Therefore, only the cross-products of the form $2ℜ(\overset{¯}{{c_{=}^{0}}^{n}}\times z_{\alpha }^{b})$, where α contains exactly two ≠, contribute at order $\delta^{2}$. The power of η that appears in this cross-product (i.e., the number of non-trivial brackets $\langle E_{0}^{i}|E_{1}^{i}\rangle$) is the number of indices *i* such that $b_{i}=1$, which is the number of steps elapsed between the two ≠. For instance, if the two ≠ happen at the ith and jth step, the contribution is

$$\begin{array}{cc} \; & 2ℜ(\overset{¯}{{c_{=}^{0}}^{n}}\times {c_{=}^{0}}^{i-1}c_{\neq }^{1}{c_{=}^{1}}^{j-i-1}c_{\neq }^{0}{c_{=}^{0}}^{n-j-1}\langle E_{0}^{i}|E_{1}^{i}\rangle \ldots \langle E_{0}^{j-1}|E_{1}^{j-1}\rangle )\\ ≃ & 2|c_{\neq }^{1}c_{\neq }^{0}\langle E_{0}^{i}|E_{1}^{i}\rangle \ldots \langle E_{0}^{j-1}|E_{1}^{j-1}\rangle |\\ ≃ & 2\eta^{j-i}V\delta^{2}. \end{array}$$

There are obviously n−k words α with exactly two ≠ separated by *k* steps, corresponding to the n−k possible choices for *i*, whose contribution is $2\eta^{k}V\delta^{2}$. Finally, the general expression for $p_{n}$ when neglecting all the higher order terms is

$$p_{n}={\left|\langle 0E_{0}^{1}\ldots E_{0}^{n}|\Psi_{n}\rangle \right|}^{2}≃1-2\left[\frac{n}{2}+\left(n-1\right)\eta +\left(n-2\right)\eta^{2}+\ldots +\eta^{n-1}\right]V\delta^{2}.$$

□

We can check the consistency of this result on two particular cases:

- If η=1, no decoherence occurs, so we recover the free evolution case during a time interval nδ instead of δ, i.e., $P_{n}=1-V{\left(n\delta \right)}^{2}$.
- If η=0, a perfect decoherence, the environment acts as an ideal measuring device, so we recover the Zeno case mentioned in the introduction, which is $p_{n}=1-nV\delta^{2}≃{\left(1-V\delta^{2}\right)}^{n}$.

Now, a Zeno effect will freeze the system in the limit of large *n* if and only if $p_{n}\underset{n\to +\infty }{⟶}1$, that is (using $\delta =\frac{T}{n}$) if $\frac{\left(n-1\right)\eta +\left(n-2\right)\eta^{2}+\ldots +\eta^{n-1}}{n^{2}}\underset{n\to +\infty }{⟶}0$. After some algebra, this expression can be simplified and leads to the following criterion:

$$\mathrm{Zeno}\;\mathrm{effect}⟺\frac{n\eta \left(1-\eta \right)+\eta \left(\eta^{n}-1\right)}{n^{2}{\left(1-\eta \right)}^{2}}\underset{n\to +\infty }{⟶}0$$

We immediately note that, if η∈[0,1) is a constant independent of *n*, the criterion is satisfied. This is natural because, as the duration of each free evolution phase goes to 0, a constant (even weak) decoherence is applied infinitely many times, so the system freezes.

We will henceforth suppose that the level of decoherence depends on *n*, with $\eta_{n}\underset{n\to +\infty }{⟶}1$. A global factor η can thus be dropped in the above criterion. Our task in the following sections will be (i) to check the criterion on some common classes of functions $\eta_{n}$ (Section 3) and (ii) to estimate the level of decoherence encountered in physical situations (Section 4).

**Remark** **1.** *How finely should the time interval be divided so that the quadratic approximation is valid? Let us forget for a moment that our system is finite dimensional and consider the Hamiltonian of a free particle P^22m, starting from the initial state $|\Psi \rangle \left(p\right)=\sqrt{\frac{\sigma }{\sqrt{\pi }\hbar }}e^{-\frac{p^{2}\sigma^{2}}{2\hbar^{2}}}$ centered around x=0 and p=0, and compute*

Var|Ψ〉(H^)=14m2〈Ψ|P^4|Ψ〉−〈Ψ|P^2|Ψ〉2=14m2∫−∞+∞p4σπℏe−p2σ2ℏ2dp−∫−∞+∞p2σπℏe−p2σ2ℏ2dp2=ℏ48m2σ4

*Hence, the quadratic approximation is valid for times shorter than tc=ℏVar|Ψ〉(H^)=22mσ2ℏ. Taking for instance $m=10^{-26}$ kg and $\sigma =10^{-10}$ m, we obtain $t_{c}=4.10^{-13}$ s. This is much shorter than the mean free time of a particle in a gas in standard conditions, which is of order $10^{-10}$ s. Thus, it seems at first sight that the decoherence steps could in practice be too separated in time for the quadratic approximation to be valid all along the free evolution step. However, decoherence does not need any actual interaction to take place (a “null measurement” is still a measurement [8]). The fact that all other surrounding particles do not interact with the particle of interest is still a gain of information for the environment, which suffices to suppress coherence with other possible histories in which they would have interacted. In this case, information is continually leaking to the environment, so it seems legitimate to divide the time interval T as finely as desired so that the quadratic approximation becomes valid, and the resulting behavior is then determined by the intensity of infinitesimal decoherence only. The philosophy of this argument is not specific to the infinite dimensional case and may be applied to our two-level system. It relies, however, on the already mentioned assumption that the strength of the interaction is more or less constant over time. This will be discussed in Section 6.*

## 3. Analytic Study of the Criterion

Whenever ${\left(n\left(1-\eta_{n}\right)\right)}_{n\in N}$ admits a limit in ${\overset{¯}{R}}_{+}\equiv R_{+}\cup \left\{+\infty \right\}$, the following lemma allows one to check immediately the criterion of the previous section.

**Lemma** **1.** *Suppose $n\left(1-\eta_{n}\right)\underset{n\to +\infty }{⟶}\alpha \in {\overset{¯}{R}}_{+}$. Then*

$$\frac{n\left(1-\eta_{n}\right)+\eta_{n}^{n}-1}{n^{2}{\left(1-\eta_{n}\right)}^{2}}\underset{n\to +\infty }{⟶}\left\{\begin{array}{c} \frac{1}{2}\;if\;\alpha =0\\ 0\;if\;\alpha =+\infty \\ \frac{1}{\alpha }+\frac{e^{-\alpha }-1}{\alpha^{2}}\;\mathrm{otherwise}. \end{array}\right.$$

**Proof.**  Let $u_{n}\equiv n\left(1-\eta_{n}\right)$. If $u_{n}\underset{n\to +\infty }{⟶}+\infty$, since $\eta_{n}^{n}-1$ is bounded, the result is immediate. If 0<α<+∞, notice that $\eta_{n}^{n}=e^{n\ln\left(1-\frac{u_{n}}{n}\right)}\underset{n\to +\infty }{⟶}e^{-\alpha }$, and rewrite:

$$\frac{n\left(1-\eta_{n}\right)+\eta_{n}^{n}-1}{n^{2}{\left(1-\eta_{n}\right)}^{2}}=\frac{1}{u_{n}}+\frac{\eta_{n}^{n}-1}{u_{n}^{2}}\underset{n\to +\infty }{⟶}\frac{1}{\alpha }+\frac{e^{-\alpha }-1}{\alpha^{2}}.$$

Finally, if α=0,

$$\begin{array}{cc} \eta_{n}^{n}=e^{n\ln\left(1-\frac{u_{n}}{n}\right)} & =e^{-u_{n}-u_{n}^{2}/2n+O\left(u_{n}^{3}/n^{2}\right)}\\ \; & =1-u_{n}-\frac{u_{n}^{2}}{2n}+O\left(\frac{u_{n}^{3}}{n^{2}}\right)+\frac{1}{2}{\left[-u_{n}-\frac{u_{n}^{2}}{2n}+O\left(\frac{u_{n}^{3}}{n^{2}}\right)\right]}^{2}+O\left(u_{n}^{3}\right)\\ \; & =1-u_{n}+\frac{u_{n}^{2}}{2}+O\left(\frac{u_{n}^{2}}{n}\right). \end{array}$$

Consequently, $\frac{n\left(1-\eta_{n}\right)+\eta_{n}^{n}-1}{n^{2}{\left(1-\eta_{n}\right)}^{2}}=\frac{u_{n}^{2}/2+O\left(u_{n}^{2}/n\right)}{u_{n}^{2}}\underset{n\to +\infty }{⟶}\frac{1}{2}.$ □

Two natural candidates for the level of short-time decoherence are $\eta_{n}=1-\frac{\alpha }{n^{\beta }}$ and $\eta_{n}=1-\alpha e^{-\beta n}$ for α,β>0. These cases can be treated by the lemma, and the different possible situations are summarized in the following table (Table 1).

## 4. Physical Considerations Concerning $\eta_{n}$

1. As previously remarked, the constant case corresponds either to the absence of decoherence (η=1) or to infinite decoherence (η∈[0,1)): these are not physically expected, except in some particular experimental setups (perfectly isolated systems for the former, and experiments specifically designed to probe the Zeno effect for the latter).
2. We have not yet introduced any duration for the decoherence step, which was considered immediate. Let us now assume that the time evolution can be divided into alternating steps dominated by either the free Hamiltonian or by the interaction Hamiltonian. The time of interaction between the system and each environment $E^{i}$, governed by H^SEi of variance Var(H^SEi)≡Vinti≡Vint (the constant strength of interaction), is still taken proportional to $\frac{T}{n}$, say equal to $\frac{cT}{n}$. This is a new assumption we make: that the time increments of both steps scale as $\frac{1}{n}$ and that the two phases can be considered on an equal footing, meaning that the two Hamiltonians are of relatively comparable strength. Then the quadratic approximation mentioned in the introduction can be applied to the whole {system + environment}. Thus, ${\left|\langle E_{\mathrm{init}}^{i}|E_{0}^{i}\rangle \right|}^{2}={\left|\langle 0E_{\mathrm{init}}^{i}|0E_{0}^{i}\rangle \right|}^{2}≃1-V_{\mathrm{int}}{\left(\frac{cT}{n}\right)}^{2}$. Moreover, since $\langle E_{\mathrm{init}}^{i}|E_{0}^{i}\rangle$ is close to the real number 1 (infinitesimal decoherence), $ℜ\left(\langle E_{\mathrm{init}}^{i}|E_{0}^{i}\rangle \right)≃\left|\langle E_{\mathrm{init}}^{i}|E_{0}^{i}\rangle \right|≃1-\frac{1}{2}V_{\mathrm{int}}{\left(\frac{cT}{n}\right)}^{2}$ is quadratic in time, similarly for $ℜ(\langle E_{\mathrm{init}}^{i}|E_{1}^{i}\rangle )$. This will also be the case for $\eta_{n}=\left|\langle E_{0}^{i}|E_{1}^{i}\rangle \right|$, because
  $$\begin{array}{cc} \sqrt{2-2|\langle E_{0}^{i}|E_{1}^{i}\rangle |}≃\sqrt{2-2ℜ(\langle E_{0}^{i}|E_{1}^{i}\rangle )}\; & =∥|E_{0}^{i}\rangle -|E_{1}^{i}\rangle ∥\\ \; & \leq ∥|E_{\mathrm{init}}^{i}\rangle -|E_{0}^{i}\rangle ∥+∥|E_{\mathrm{init}}^{i}\rangle -|E_{1}^{i}\rangle ∥\\ \; & =\sqrt{2-2ℜ(\langle E_{\mathrm{init}}^{i}|E_{0}^{i}\rangle )}+\sqrt{2-2ℜ(\langle E_{\mathrm{init}}^{i}|E_{1}^{i}\rangle )}\\ \; & ≃2\sqrt{V_{\mathrm{int}}}\frac{cT}{n}, \end{array}$$
  so $\eta_{n}=\left|\langle E_{0}^{i}|E_{1}^{i}\rangle \right|≳1-2V_{\mathrm{int}}{\left(\frac{cT}{n}\right)}^{2}$ is also at least quadratic. Said differently, *because quantum mechanical short time evolutions are always quadratic, and this is true also for the environment’s evolution, infinitesimal steps of decoherence induced on a system by its surrounding environment are likely to be of the form $\eta_{n}=1-\frac{\alpha }{n^{\beta }}$ with β≳2. This could constitute a universal reason why the universe is not frozen by the quantum Zeno effect.*
  An example of such an interaction is the following. Consider that the system is a qbit in the state $\frac{|0\rangle +|1\rangle }{\sqrt{2}}$, and the environment is a particle initially centered around x=0 with momentum $p_{0}$, that is $|\Psi_{0,p_{0}}\rangle =\frac{1}{\sqrt{\sqrt{\pi }\sigma }}e^{ip_{0}x}e^{-\frac{x^{2}}{2\sigma^{2}}}\in L^{2}\left(R\right)$. The system’s state is recorded on the (|0〉,|1〉) basis due to the interaction H^SE=vσz^⊗P^ so that, after some time $\delta \propto \frac{1}{n}$,
  $$\left\{\begin{array}{c} |0\rangle |\Psi_{0,p_{0}}\rangle ⟶|0\rangle |\Psi_{v\delta ,p_{0}}\rangle \\ |1\rangle |\Psi_{0,p_{0}}\rangle ⟶|1\rangle |\Psi_{-v\delta ,p_{0}}\rangle , \end{array}\right.$$
  where
  $$\eta_{n}=\left|\langle \Psi_{v\delta ,p_{0}}|\Psi_{-v\delta ,p_{0}}\rangle \right|=\left|\frac{e^{2ip_{0}v\delta }}{\sqrt{\pi }\sigma }\int_{R}e^{-\frac{x^{2}+{\left(v\delta \right)}^{2}}{\sigma^{2}}}dx\right|=e^{-\frac{{\left(v\delta \right)}^{2}}{\sigma^{2}}}≃1-\frac{v^{2}}{\sigma^{2}}\delta^{2},$$
  so this interaction induces indeed a short time quadratic decoherence as long as the increment of time satisfies $\delta \ll \frac{v}{\sigma }$.
3. What if the assumption of comparable strengths of the Hamiltonians fails, for instance, if the free evolution term is negligible compared to the coupling with the environment? This amounts to taking *c* or $V_{\mathrm{int}}⟶+\infty$ and thus lifting the quadratic approximation for the interaction Hamiltonian. A possibility then is to consider that $|E_{0}^{i}\left(t\right)\rangle$ and $|E_{1}^{i}\left(t\right)\rangle$ follow two independent Brownian motions starting in $|E_{\mathrm{init}}^{i}\rangle$ on the sphere of all possible states in $H_{E^{i}}$ during the duration δ of the decoherence step. If the latter exceeds the typical time of diffusion on the sphere, we recover the case of a constant η∈[0,1) (infinite decoherence, case n°1 above) with $\eta ∼\frac{1}{\sqrt{\dim(H_{E^{i}})}}$, as shown in [9]. If it is shorter than the diffusion time (but still longer than the quadratic regime), $|E_{0}^{i}\left(\delta \right)\rangle$ lies in the vicinity of $|E_{\mathrm{init}}^{i}\rangle$ on the sphere, which is approximately a ball. It is well known that the typical length of diffusion is expressed as $∥|E_{\mathrm{init}}^{i}\rangle -|E_{0}^{i}\left(\delta \right)\rangle ∥≃D\sqrt{\delta }$, which implies $\left|\langle E_{\mathrm{init}}^{i}|E_{0}^{i}\left(\delta \right)\rangle \right|≃\sqrt{1-{\left(D\sqrt{\delta }\right)}^{2}}≃1-\frac{D^{2}}{2}\delta$. If δ is still taken $\propto \frac{1}{n}$, we are now in the intermediate regime studied above, with β=1 and $\alpha \propto D^{2}$. This corresponds to situations where the system’s evolution is slowed down because of its monitoring by the environment. In the limit of strong interaction, the diffusion constant *D* will go to infinity and we recover the Zeno effect, whereas a weak interaction tends to the free evolution case.

## 5. Comparison with Presilla et al.’s Continuous Model

The situation explored by Presilla, Onofrio, and Tambini in Section IV of [4] is very similar to ours—a two-level system undergoing some external monitoring in addition to its free evolution—but the major difference relies in the continuous nature of the model. Precisely, they solve the Lindblad’s master equation governing the competition between the system’s internal evolution in the presence of a resonant field (producing Rabi oscillations between levels 1 and 2) and a continuous probing of the occupancy of level 1, whose intensity can modulated by a factor κ(t). The equation, which can in this case be solved analytically, reads

dρ(t)dt=−iℏH^(t),ρ(t)−12κ(t)A^,A^,ρ(t),

where A^ is the monitored observable.

**Figure 2:**
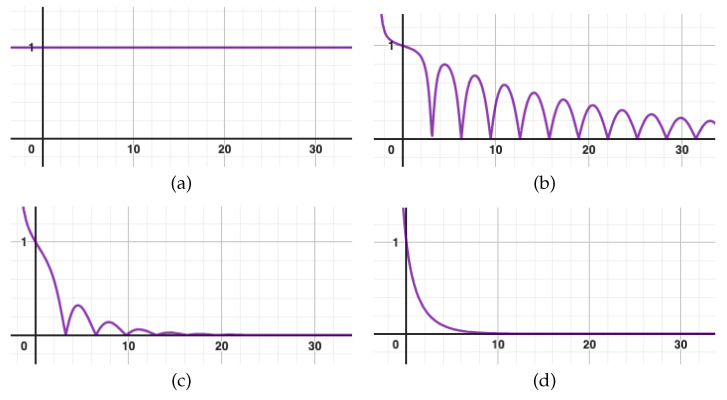
Plot of η(t) for different values of κ. (**a**) κ=0. (**b**) κ=0.2. (**c**) κ=1. (**d**) κ=5.

It is interesting to check whether their findings are compatible with our model, in particular to determine the level of short time decoherence η and the parameter β that correspond to their situation and see if our conclusions still apply. To do so, recall that the density matrix of a system entangled with an environment in a state $|\Psi \rangle =c_{1}|1\rangle |E_{1}\rangle +c_{2}|2\rangle |E_{2}\rangle$ is given by $\rho =\left(\begin{array}{cc} {\left|c_{1}\right|}^{2} & c_{1}\overset{¯}{c_{2}}\langle E_{2}|E_{1}\rangle \\ \overset{¯}{c_{1}}c_{2}\langle E_{1}|E_{2}\rangle & {\left|c_{2}\right|}^{2} \end{array}\right)$. Therefore, although the environment’s evolution is not specified in Presilla et al.’s model, one can still deduce $\left|\langle E_{1}|E_{2}\rangle \right|$, and in particular its short-time behavior corresponding to our η, by computing the quantity $\eta \left(t\right)=\frac{\left|\rho_{12}\left(t\right)\right|}{\sqrt{\rho_{11}\left(t\right)\left(1-\rho_{11}\left(t\right)\right)}}$. Using the expressions (84) and (85) for $\rho_{11}\left(t\right)$ and $\rho_{12}\left(t\right)$ given in [4], we can plot η(t) for different values of the parameters. In Figure 2, we show η(t) for the initial conditions $\rho_{11}\left(0\right)=1$, $\rho_{12}\left(0\right)=\rho_{21}\left(0\right)=0$, corresponding to a system starting in the pure state |1〉, and different values of κ (we have set the Rabi frequency $\omega_{R}=1$, so that we actually plot in units of $\frac{\kappa }{\omega_{R}}$ and $\omega_{R}t$).

Crucially, we see that η(t) has a non-vanishing first derivative in 0 (except in the extreme case κ=0, where of course no decoherence occurs). When plotting the latter as a function of κ, we observe that $\eta^{\prime }\left(0\right)=-\frac{\kappa }{2}$. This means that the short-time decoherence is of the form $\eta \left(\delta \right)≃1-\frac{\kappa }{2}\delta$, which corresponds to the case β=1 and $\alpha =\frac{\kappa }{2}$ in our model (recalling that $\delta =\frac{T}{n}$ and equating $T=1=\omega_{R}^{-1}$ as the characteristic times of the two models).

Are the two models consistent? According to the table of Section 3, we expect an intermediate regime between Zeno freezing and free evolution, where the system is still evolving but slowed down. This is indeed what happens in Presilla et al.’s model. Importantly, they conclude that “strong Zeno inhibition [κ→∞ limit] as well as full Rabi oscillations [κ→0 limit] are two trivial extreme regimes. However, [their model] tells us that another interesting and unexplored regime exists. It is the regime which occurs when the measurement coupling is comparable to the critical value. In this case a strong competition between stimulated transitions and measurement inhibition takes place.”

We can even be more precise and compare quantitatively the slowing down of the system as a function of $\alpha =\frac{\kappa }{2}$. In our model, the quadratic evolution term $1-VT^{2}$ is replaced by $1-2\left(\frac{1}{\alpha }+\frac{e^{-\alpha }-1}{\alpha^{2}}\right)VT^{2}$, so that the time unit is effectively replaced by

$$T⇝T_{\mathrm{eff}}=\frac{T}{\sqrt{2\left(\frac{1}{\alpha }+\frac{e^{-\alpha }-1}{\alpha^{2}}\right)}}=\frac{T}{2\sqrt{\frac{1}{\kappa }+2\frac{e^{-\kappa /2}-1}{\kappa^{2}}}}.$$

In the continuous model, the proper frequency $\omega_{R}$ of the system becomes under monitoring $\sqrt{\omega_{R}^{2}-\frac{\kappa^{2}}{16}}$, so that the time unit is effectively replaced by

$$T⇝T_{\mathrm{eff}}=\frac{T}{\sqrt{1-\frac{\kappa^{2}}{16}}}.$$

Of course, these two correction factors are not equal, but they turn out to coincide quite well, at least in the small κ case. For instance, on the whole range κ∈[0,2.5], they deviate by no more than 5% to each other, as can be seen by plotting the function $F:x\mapsto \frac{2\sqrt{\frac{1}{x}+2\frac{e^{-x/2}-1}{x^{2}}}}{\sqrt{1-\frac{x^{2}}{16}}}$. For larger κ, though, the two models completely disagree at the quantitative level. This may be due to the fact that our model, by considering free evolution steps of finite time but immediate steps of decoherence, implicitly assumes some relatively weak coupling with the environment. In particular, it is unable to account for the critical transition that happens at κ=4.

As a final comment in this section, it is interesting to remember that a continuous Lindblad’s master equation corresponds to the intermediate regime β=1. This does not necessarily affect the argument given in Section 4 to justify that the most typical physical situation might be β≳2. Indeed, although Lindblad’s equation is somehow universal (being the most general equation governing an open quantum system interacting with a Markovian environment), picking a preferred observable A^ is not. In reality, the mutual monitoring between all the subsystems in the universe arise from complex interactions between a huge number of particles, and which observables are more recorded than the others is far from obvious (this discussion is related to the famous preferred-basis problem, see [10] for an updated bibliography).

## 6. Discussion

We have presented a model designed to check whether quantum mechanics indeed predicts that the universe should evolve. To remain as universal as possible, no specific form of Hamiltonian was assumed. It allowed us to determine the level of decoherence (induced by a surrounding environment) needed to freeze a two-level quantum system, arguably the kind of system most prone to the Zeno effect. We have found that if, during a time interval $\frac{T}{n}$, the environment distinguishes between the two states according to $\left|\langle E_{0}^{i}|E_{1}^{i}\rangle \right|≃1-\frac{\alpha }{n^{\beta }}$ with β>1, then free evolution wins over decoherence, and the system is not frozen. In the most generic case, because quantum mechanical short time evolutions are always quadratic (and this is true for the system as well as for the pair {system + environment}), we find β≳2; hence, the universe is indeed not frozen. We have finally made a quantitative comparison with the continuous master equation model by Presilla, Onofrio and Tambini [4]. The links between the two models are non-trivial but we have found a good agreement at least in the low coupling regime.

The main weaknesses of the model, leading to possible improvements, are the following.

- Is the discrete setup legitimate? A succession of infinitesimal steps is not necessarily the same as a joint continuous evolution.
- What happens if the coupling with the environment is not supposed roughly constant anymore? Mathematically, this means that the $\eta^{i}$ values are not equal, and the infinitesimal decoherence rate (i.e., the flow of information) at time *t* could be modeled in the limit n⟶+∞ as a continuous quantity 1−dη(t). It is natural to ask for the set of such functions that entail a Zeno freezing. Furthermore, the durations of the steps could also be non-constant (like following a Poisson process, as done in [11]).
- That the environments $E^{i}$ are distinct and non-entangled is a very unphysical assumption. In some cases, previous entanglement among the environments can dramatically change the efficiency of decoherence. As an example, take an environment composed of two qbits called $E^{1}$ and $E^{2}$ initially maximally entangled; the system interacts with $E^{1}$ and then with $E^{2}$ via a C-NOT gate:
  $$\begin{array}{cc} \underset{S}{\underset{︸}{\frac{1}{\sqrt{2}}\left(|0\rangle +|1\rangle \right)}}⊗\underset{E^{1}+E^{2}}{\underset{︸}{\frac{1}{\sqrt{2}}\left(|00\rangle +|11\rangle \right)}}\; & \;\underset{C-NOT_{SE^{1}}}{⟶}\;\underset{\rho_{S}=\left(\begin{array}{cc} \frac{1}{2} & 0\\ 0 & \frac{1}{2} \end{array}\right)\;\text{:}\;S\;\mathrm{is}\;\mathrm{perfectly}\;\mathrm{decohered}}{\underset{︸}{\frac{1}{2}\left(|000\rangle +|011\rangle +|110\rangle +|101\rangle \right)}}\\ \; & \;\underset{C-NOT_{SE^{2}}}{⟶}\;\frac{1}{2}\left(|000\rangle +|011\rangle +|111\rangle +|100\rangle \right)\\ \; & \;=\underset{\rho_{S}=\left(\begin{array}{cc} \frac{1}{2} & \frac{1}{2}\\ \frac{1}{2} & \frac{1}{2} \end{array}\right)\;\text{:}\;\mathrm{coherence}\;\mathrm{has}\;\mathrm{revived}}{\underset{︸}{\frac{1}{\sqrt{2}}\left(|0\rangle +|1\rangle \right)⊗\frac{1}{\sqrt{2}}\left(|00\rangle +|11\rangle \right).}} \end{array}$$

## Figures and Tables

**Table 1:** Possible decoherence regimes.

$\eta_{n}$	Regime	$\lim_{n\to +\infty }p_{n}$
1	Free evolution	$1-VT^{2}$
Constant ∈[0,1[	Zeno effect	1
$1-\frac{\alpha }{n^{\beta }}$ with β∈]0,1[	Zeno effect	1
$1-\frac{\alpha }{n^{\beta }}$ with β>1	Free evolution	$1-VT^{2}$
$1-\frac{\alpha }{n}$	Intermediate	$1-\underset{\begin{array}{c} \underset{\alpha \to +\infty }{⟶}0\;\text{:}\;\mathrm{Zeno}\;\mathrm{effect}\\ \underset{\alpha \to 0}{⟶}1\;\text{:}\;\mathrm{free}\;\mathrm{evolution} \end{array}}{\underset{︸}{2(\frac{1}{\alpha }+\frac{e^{-\alpha }-1}{\alpha^{2}})}}VT^{2}$
$1-\alpha e^{-\beta n}$	Free evolution	$1-VT^{2}$

## Data Availability

The original contributions presented in this study are included in the article. Further inquiries can be directed to the corresponding author.
